# Plasmon-Induced Enhanced Light Emission and Ultrafast Carrier Dynamics in a Tunable Molybdenum Disulfide-Gallium Nitride Heterostructure

**DOI:** 10.3390/ma15217422

**Published:** 2022-10-22

**Authors:** Yuba Poudel, Sairaman Seetharaman, Swastik Kar, Francis D’Souza, Arup Neogi

**Affiliations:** 1 Department of Physics, University of North Texas, Denton, TX 76203, USA; 2 Department of Chemistry, University of North Texas, Denton, TX 76203, USA; 3 Department of Physics, Northeastern University Boston, Boston, MA 02115, USA; 4 Institute for Fundamental and Frontier Sciences, University of Electronic Science and Technology, Chengdu 610056, China

**Keywords:** nanoplasmonics, ultrafast spectroscopy, gallium nitride, hybrid 2D/3D semiconductor, molybdenum disulfide

## Abstract

The effect of localized plasmon on the photoemission and absorption in hybrid molybdenum disulfide-Gallium nitride (MoS_2_-GaN) heterostructure has been studied. Localized plasmon induced by platinum nanoparticles was resonantly coupled to the bandedge states of GaN to enhance the UV emission from the hybrid semiconductor system. The presence of the platinum nanoparticles also increases the effective absorption and the transient gain of the excitonic absorption in MoS_2_. Localized plasmons were also resonantly coupled to the defect states of GaN and the exciton states using gold nanoparticles. The transfer of hot carriers from Au plasmons to the conduction band of MoS_2_ and the trapping of excited carriers in MoS_2_ within GaN defects results in transient plasmon-induced transparency at ~1.28 ps. Selective optical excitation of the specific resonances in the presence of the localized plasmons can be used to tune the absorption or emission properties of this layered 2D-3D semiconductor material system.

## 1. Introduction

The hybrid integration of a 2D-3D semiconductor enabled by the exceptional lattice match between molybdenum disulfide and gallium nitride semiconductors offers an ideal platform for realizing a tunable gap material system for broadband light emission and photovoltaics [1]. Gallium nitride has a wide bandgap with band-edge states in the ultraviolet and defect-induced bandgap states in the green/yellow [2,3] wavelength regime. A single or few monolayers of MoS_2_ exhibit direct excitonic transitions in the red wavelength [4]. The hybrid integration of monolayer MoS_2_ on bulk GaN has shown PL emission over a broad range of the visible spectrum covering red, yellow, and ultraviolet wavelengths [5]. The hybrid integration of this material system results in an increased density of states within the bandgap energy regime of GaN due to an overlap of the high-density electronic state of MoS_2_ with the defect bands in GaN [6]. However, the relatively low light absorption in the monolayer thin MoS_2_ layer in the hybrid 2D-3D semiconductor system impedes the development of efficient broadband absorbers. The relatively low carrier density in a monolayer of MoS_2_ also produces a relatively weak emission efficiency despite a strong exciton binding energy. Additional measures must be explored to increase the carriers available for light generation in the red wavelength regime.

Recently nano-plasmonic effects have proven to be an effective means for enhancing photocurrent generation [7] or absorption for sensing [8], tunable antennae [9], and tuning the wavelength of absorption and emission [10]. Metal nanoparticles hybridized with 2D materials or graphene can modulate the absorption of the quantized states [11,12] in these materials, leading to coherence effects due to light-matter interaction. The plasmonic properties of the nanoparticles can be tailored by the dielectric environment both within and outside the nanoparticles [13]. Gallium nitride is an elastic medium with strong elastic and built-in piezoelectric strain [2,14]. The near-field coupling of light due to nanoplasmonics with the coherent phonons in the nitride system that can occur in the ultrafast domain can also lead to an interesting material system in plasmomechanics [15].

In this work, two different localized plasmon systems were designed to be coupled to specific energy states of the hybrid molybdenum disulfide-gallium nitride (MoS_2_-GaN) semiconductor system. The influence of these localized plasmons on the modulation of the absorption in the MoS_2_-GaN semiconductor system depends on the overlap of energy of the localized plasmon resonances and the specific absorption states within the hybrid semiconductor system. Localized surface plasmons due to platinum (Pt) nanoparticles with their ultraviolet plasmon energy resonant to the bandgap of GaN are ideal for modifying the band edge absorption and enhancing the spontaneous emission rate in GaN. The plasmon energy due to Pt is also resonant to the C-exciton state of the MoS_2_ and can influence carrier transport. These continuum absorption states in the 2D material can also be modified due to the Pt nanoparticles. For manipulating the MoS_2_ layer, Au nanoparticles with their localized surface plasmon energy resonant to the A and B exciton states in MoS_2_ is another material system studied in this work. The resonant coupling of Au NPs with the defect bands of GaN can also lead to absorption change and energy transfer from the defect band of GaN.

The trapping of the carriers by the defects in GaN changes the diffusion length and mobility, which critically influences device efficiencies [16]. The relaxation and recombination of the photogenerated carriers in the band-edge, defect, and excitonic states of the MoS_2_-GaN semiconductor system can be modulated through the photonic density of states (PDS) of the localized plasmons. We have thereby used transient differential absorption spectroscopy to study the carrier scatter and the lifetime of the carriers in the excited state of the plasmonic MoS_2_-GaN system to evaluate the optoelectronic properties of this novel material system.

## 2. Materials and Methods

GaN film was grown by chemical vapor deposition on a double-sided polished sapphire substrate at 1150 °C. The thickness of the Si: doped GaN film was 4.5 μm. The 2D layered material was formed by evaporating the commercially available MoS_2_ nanoflakes (Graphene supermarket) (1–8 layers) in water on the CVD-grown GaN layer. The plasmonic nanostructures of Au and Pt were formed on the MoS_2_-GaN template by thermal evaporation of commercially available Au and Pt nanoparticles in solution with an average diameter of about 80 nm and 70 nm resp (Nanocomposix) at a temperature of 300 °C for 30 min.

**Figure 1:**
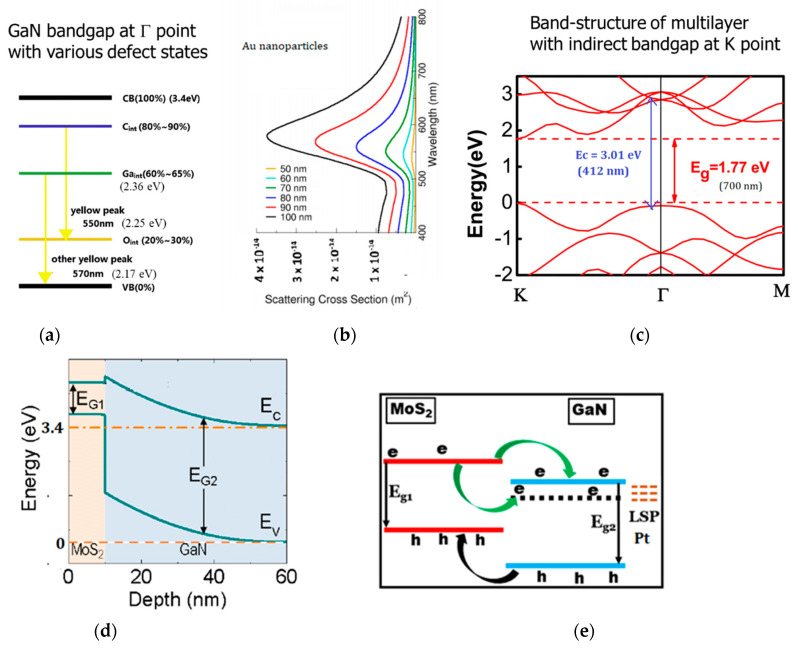
Schematic showing the band line up of hybrid MoS_2_-GaN hybrid semiconductor system. (**a**) GaN bandgap at the gamma point with Gallium interstitial, oxygen, and interstitial carbon defect leading to emission in the yellow or greenish yellow wavelength range [17]. (**b**) also shows the scattering cross-section of Au nanoparticles with varying diameters having resonant plasmon energy corresponding to the defect states of GaN. (**c**) The band structure of multilayer MoS_2_ depicts an indirect absorption edge at the K point [18] with energy that overlaps with the plasmon energy of Au nanoparticles shown in Figure 2. There is also an enhanced electronic density of states at the Г point at 3.01 eV (**d**). The band lineup of the hybrid semiconductor is a type II heterojunction [19]. The radius of the platinum nanoparticles is chosen to have plasmon energy corresponding to the direct bandgap of the GaN. It may also overlap with the enhanced density states at the Г point of the MoS_2_. (**e**) The possible carrier transfer process in Pt-based GaN-MoS_2_ hybrid system with the indirect band lineup.

**Figure 2:**
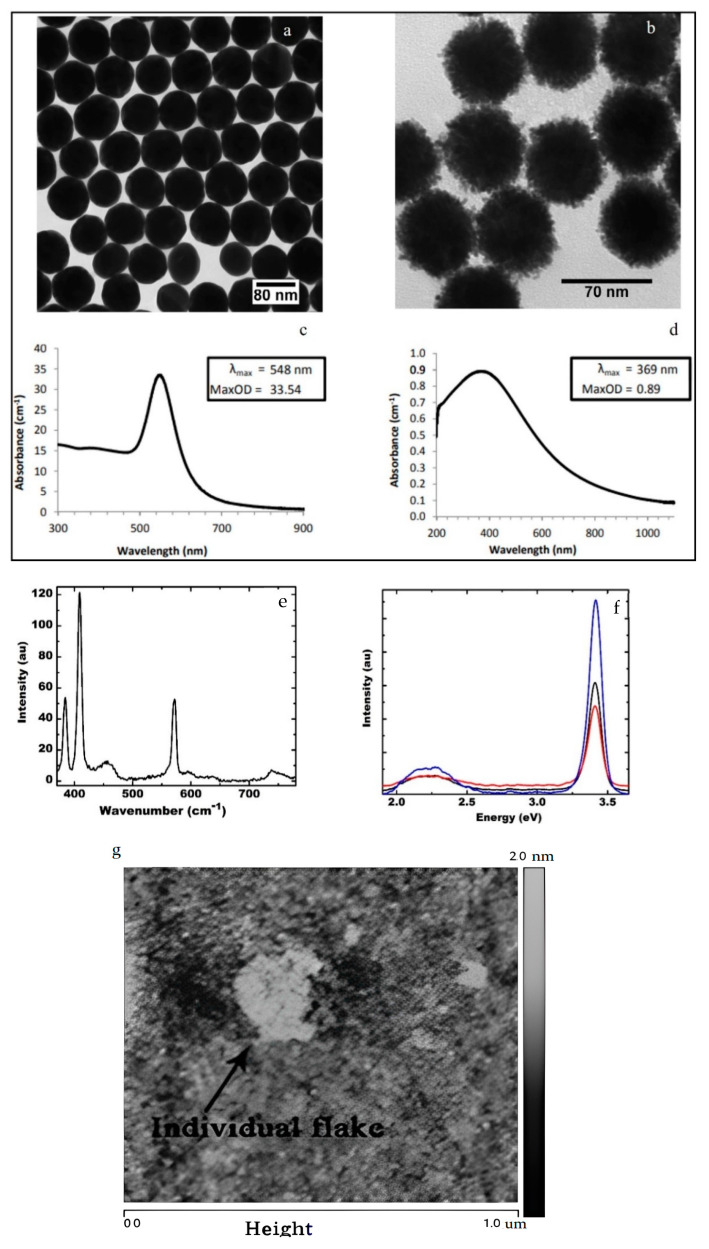
(**a**) SEM Images of Au NPs. (**b**) SEM images of Pt NPs. (**c**) The absorption spectrum of Au NPs. (**d**) The absorption spectrum of Pt NPs. (**e**) Raman characteristics show the active Raman modes in GaN and MoS2. (**f**) PL emission from GaN showing the band-edge emission and defect band emission showing enhanced band-edge and defect band PL emission intensity with Pt plasmons (blue) and quenching of PL emission intensity with Au plasmons (red) with respect to MoS_2_-GaN (black). (**g**) AFM image of MoS_2_ flakes used in this experiment.

The optical properties of the MoS_2_-GaN semiconductor were reported in our previous study. Figure 1a shows a schematic of GaN bandstructure with deep-level defects that lead to yellow or green emissions in this wide bandgap semiconductor system [17]. Figure 1c shows the band structure of multilayer MoS_2_ [18]. It can be observed that the indirect absorption energy levels in the multilayer MoS_2_ are lower than the interstitial defect levels states in GaN, providing the possibility of additional carrier relaxation channels. MoS_2_ semiconductors also have enhanced electron density of states at the Г point at 3.01 eV, closer to the exciton absorption states of bulk GaN. The interface of the hybrid semiconductor system results in a type II heterojunction estimated from DFT calculations [5]. The hybrid GaN/MoS_2_ semiconductor has a conduction band offset of 0.23 eV [19]. The diameter of metal nanoparticles can be selected to be tuned to have plasmon energy resonant to the electronic absorption of the hybrid semiconductors. As shown in the simulated scattering cross-section of Au- nanoparticles, the electromagnetic field at the Au-plasmon resonance can be coupled to the defect emission and lead to the excitation of carriers above the indirect band states in MoS_2_. The particle size dependence of Au-plasmon peaks estimated in free space and the experimentally measured plasmons in a solvent are shown in Figure 2a. However, the aggregation of nanoparticles and the dielectric environment of MoS_2_ around Au-NPs can lead to a significant red shift in the plasmon resonance due to screening [20]. On the other hand, the Pt nanoparticles have higher plasmon energy in the ultraviolet wavelength, resulting in interband carrier transition in GaN semiconductors. The size of the Pt nanoparticles used in this work is shown in Figure 2b. The plasmon energy corresponding to 369 nm is very close to the exciton absorption states in GaN that exhibit stable room-temperature excitonic absorption at 20 meV below the interband absorption state at 3.4 eV. The approximated band lineup in the presence of the Pt-based plasmon is shown in Figure 1e. The Pt nanoparticles can also excite carriers to the C-exciton absorption with the enhanced density of states at the Г point of MoS_2_. Due to the lack of direct bandgap in a multilayer system, there is no emission in MoS_2_. However, the absorption states do exist, leading to the probability of interband carrier transition. These carriers excited above the bandgap in GaN or the enhanced absorption states of MoS_2_ at the c-band due to enhanced density of states have multiple decay channels due to the presence of the defect states, excitonic states, and the plasmonic states, which can result in a very fast and efficiency carrier density modulation. It can result in ultrafast modulation of the absorption characteristics of the semiconductor dependent on the role of the plasmonic resonances. These plasmon resonances can be selectively switched on or off by choosing the excitation wavelength. For example, by choosing a visible excitation light source, the Pt nanoparticle-induced plasmons can be switched off, and the Au nanoparticles play the role of electromagnetic coupling.

The steady-state absorption spectra at room temperature were measured using a spectrophotometer with a white-light source and a photomultiplier detector. The PL measurements were performed using a homebuilt micro-photoluminescence spectroscopy setup. A Helium Cadmium laser was used as the excitation source with a UV excitation line at 3.82 eV to study the emission from the GaN band-edge and defect states. The indirect band lineup of the heterojunction does not result in any emission from the C excitons of MoS_2_ or the A and B excitons when the excitation energy is in the continuum states using the 3.82 eV source. The emission from the MoS_2_ multilayers was not studied in this work, as the indirect nature of the MoS_2_ does not emit any light. However, the transient absorption characteristics were studied as the plasmons significantly modify the absorption characteristics in the ultrafast time domain. This modulation depends significantly on the energy of optical excitation. The micro-Raman spectrum was measured using an Olympus BX51 microscope with optical excitation of 2.33 eV. The time-resolved PL measurements were performed using an 80 MHz laser, 80 fs Ti: Sapphire laser oscillator source with an optical excitation at 2.8 eV was used. A 100 fs Ti: Sapphire amplifier was used as a pump source for the differential transmission measurements, with a white light continuum generated from the amplifier as the probe source. Two different excitation energies were used in this study. A high-energy excitation line of the OPA was used at 3.54 eV to study the relaxation of the carriers from the continuum states of GaN or C-excitons in MoS_2_. The Au nanoparticles cannot be excited directly by laser excitation at this excitation wavelength. However, the generation of defect emission within GaN due to the above bandgap absorption in GaN can result in a secondary excitation of the plasmons due to Au nanoparticles. The defect emission can be avoided by below bandgap excitations. An excitation line at 3.06 eV was used to avoid the interband excitation in GaN and observe the transient absorption change of the A and B excitons in MoS_2_ and the defect states in GaN with the white-light continuum probe. The difference in absorption of the probe pulses (ΔA) in the presence and absence of pump pulses is measured at different delay times by varying the distance traveled by the pump and probe pulses.

## 3. Results and Discussion

The Gold (Au) and Platinum (Pt) nanospheres procured from Nanocomposix have a diameter of about 80 nm and 70 nm, respectively. Their corresponding plasmon energy (Figure 2a,b) is centered at 2.26 eV (548 nm) (Figure 2c) and 3.26 eV (369 nm) (Figure 2d), respectively. The Raman spectrum of MoS_2_-GaN heterostructure is shown in Figure 2e. The active Raman modes in MoS_2_—$E_{2g}^{1}$ and $A_{1g}$ are observed at 384 cm^−1^ and 408 cm^−1^, respectively, showing the dominance of the few-layer structures (Figure 2e) [21]. The Raman modes centered at 572 cm^−1^ and 738 cm^−1^ and represent, respectively, the $E_{2}$ and $A_{1}$ longitudinal optical (LO) phonon mode of the GaN layer [22]. From the Raman spectrum, the layer number of MoS_2_ flakes varies between 2 to 8 layers, and hence the thickness of MoS_2_ varies between 0.75 nm to about 6 nm. The lateral dimension of the MoS_2_ flakes is a few hundred nm. The PL spectrum of GaN shows a band-to-band transition centered at 3.4 eV and a weak but broad defect band luminescence centered at 2.26 eV (Figure 2f). The steady-state absorption spectrum (Supporting Figure S1) shows the absorption bands in MoS_2_ and GaN. It should be noted that the defect emission band is observed only when the optical excitation is above the GaN band edge. Thus one can selectively excite the Au plasmons and populate the MoS_2_ states without populating the defect levels within GaN by choosing an excitation wavelength below the GaN bandedge around 3.06 eV. The photoexcited carriers relax through carrier-carrier scattering or electron-phonon scattering from the conduction band of GaN to the defect states, which are relatively longer-lived [2]. For excitation below the bandgap of GaN, there is a lack of appreciable carrier density available in the defect state for any emission. Thus the 3.54 eV optical pump is used in the ultrafast transient spectroscopy to induce an active defect emission.

A comparison of the PL spectrum, measured using a 3.82 eV CW laser, exhibits an enhancement of the PL emission of the GaN bandedge due to the Pt nanoparticles, whereas a quenching of the emission due to Au nanoparticles (Figure 2e). It is due to the increase in the radiative recombination rate in the presence of resonant plasmons due to Pt nanoparticles. As the Au nanoparticles have a localized surface plasmon (LSP) energy below the bandgap, there is no change in the spontaneous emission due to the plasmons. Instead, the dissipative effect due to the plasmons results in emission quenching at room temperature. The quenching in the bandedge emission at the UV emission due to the dissipation induced by Au nanoparticles is similar to that observed in bulk ZnO [23].

**Figure 3:**
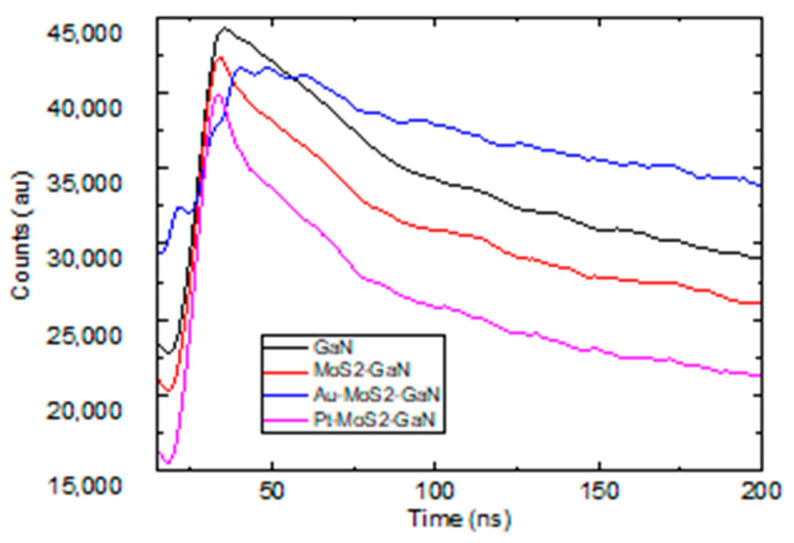
Time-resolved photoluminescence decay time of the defect band showing slower recombination LT with Au plasmons (blue) and faster recombination LT with Pt plasmons. The black and red curves represent the TRPL characteristics of GaN and MoS_2_-GaN, respectively.

Figure 3 shows the time-resolved photoluminescence measurements of the carrier recombination from the defect state. The radiative lifetime of the carriers in the defect state of the MoS_2-_ GaN system is reduced owing to the resonant coupling of the localized plasmon due to Pt nanoparticles with the excited photocarriers in the bandedge states of GaN. As Figure 2d shows, the localized plasmon energy of Pt overlaps with the bandgap of MoS_2_-GaN or GaN semiconductor systems (Figure 1a,c). Despite the long lifetime of the defect level defect states as observed from the bare GaN sample, the carrier transfer to the exciton states in MoS_2_ also reduced the PL lifetime of the defect emission in the MoS_2_-GaN heterojunction. In contrast, the PL lifetime of the defect state is increased due to Au nanoparticles. The overlap of the plasmon energy due to Au NPs with the defect states provides additional pathways for the carriers to decay to the A and B exciton states of MoS_2_ before recombination with the holes in the ground state of GaN and results in an enhanced PL lifetime. The reduced (enhanced) lifetime increases (decreases) the total number of carriers available for optical excitation in the ground state and an enhanced PL emission of the bandedge emission in the presence of Pt nanoparticles. The magnitude of the rate of change of the spontaneous emission due to the Pt plasmons is less than the enhancement of the magnitude of bandedge emission intensity. It occurs as a part of the photogenerated carriers at the UV wavelength (3.4 eV), including the defect emission in GaN reabsorbed within the MoS_2_ film.

**Figure 4:**
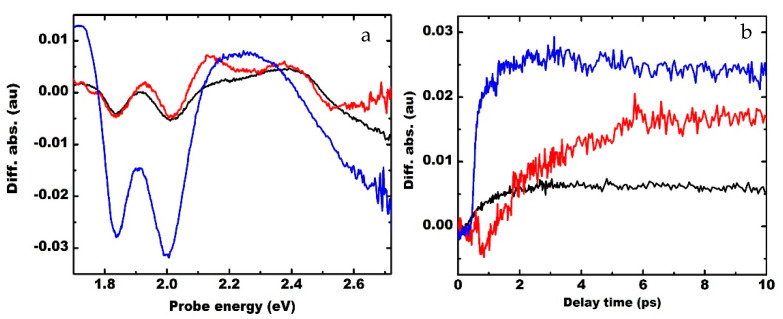
(**a**) Ultrafast absorption spectrum of MoS_2_ on quartz with 3.54 eV pump, (black), MoS_2_ on GaN with 3.54 eV pump, (red) and MoS_2_ on GaN with 3.06 eV pump, (blue) showing the excitonic absorption state and photoinduced absorption bands at 1 ps with 10 microjoule excitation energy density. (**b**) Decay kinetics showing the recovery of probe absorption for MoS_2_ on GaN (black), MoS_2_ on GaN with Au plasmons (red), and MoS_2_ on GaN with Pt plasmons (blue) at the defect band of GaN (2.26 eV).

GaN is a UV absorber, MoS_2_ has an absorption in the UV and visible region, and the heterostructure formed from these semiconductors is a broadband absorber [5]. Due to the indirect nature of the optical transitions in the multilayer MoS_2_ on GaN, the plasmon-induced effects cannot be investigated using time-resolved photoluminescence spectroscopy. Transient absorption spectroscopy with selective photo-excitation above and below the GaN bandedge can explain the carrier transfer and plasmon-induced localized excitation effects. Figure 4a shows the effect of the GaN substrate on the absorption in the MoS_2_ film with a quartz substrate as a reference. It shows the transient spectra for the defect states and the exciton states in MoS_2_ at 1 ps for a certain power density of the excitation light. Excitation at 3.54 eV induces the interband transition in both species (MoS_2_ and GaN); however, the excitation at 3.06 eV is resonant to the C excitonic state of MoS_2_ and does not excite the interband transition in GaN. The transient absorption spectrum of MoS_2_ on quartz substrate measured at a delay time of about one ps shows the negative amplitude bands, called photobleaching (PB) bands, at the A and B excitonic absorption states. [24] There are also the observed positive amplitude bands, termed the photoinduced absorption at other energy states (Figure 4a). The formation of PB bands is attributed to the decrease in absorption of the probe due to the formation of excitonic states due to an optically excited pump pulse with excitation energy above the GaN bandedge. The increase in absorption is attributed to the absorption of the probe pulses by the excited carriers due to the pump pulse. For MoS_2_ on a quartz substrate, the A and B excitonic states appear at 1.84 eV and 2.01 eV, respectively. A broad photoinduced absorption band appears at an energy higher than B exciton at a longer delay time and saturates at a delay time of about 5 ps. For MoS_2_ on GaN substrate, a significant modification in the photoinduced absorption band is observed with optical excitation of 3.54 eV. The photoinduced absorption band is separated into two bands due to a decrease in absorption of probe pulse over a certain band centered at 2.26 eV. However, with an optical excitation at 3.06 eV, there is no remarkable difference in the photoinduced absorption in the presence of the GaN layer as the defect states are depopulated due to enhanced photonic density of states due to the plasmons. In addition, no decrease in absorption of the probe pulse is observed when the GaN layer without MoS_2_ is optically excited with interband GaN excitation. Thus, the modification of the photoinduced absorption is attributed to the defect band of GaN, which is activated with the 3.54 eV excitation. Strong photobleaching is observed above 2.7 eV, which is more for the 3.06 eV excitation. It presumably occurs due to the resonant excitation of carriers by the pump to the interband states in MoS_2_ around the G-band that has a large density of states which is enhanced due to the screening induced by the GaN dielectric substrate.

Figure 4b shows the transient absorption at 2.26 eV, which corresponds to the defect states of GaN. The optically excited carriers in both GaN and MoS_2_ can be trapped at the defect band of GaN. The relaxation of carriers trapped at the GaN defect band is modulated by LSP plasmons due to metal nanoparticles (NPs). This band is saturated at a delay time of about 2.8 ps, and the recovery of probe absorption at this subband energy state occurs at a very slow rate. The slow recovery of absorption of the probe is attributed to the trapping of carriers at longer-lived defect states. It shows that the recovery time in the presence of the LSP due to Pt NPs is the fastest, resulting in faster recycling of the carriers and leading to PL enhancement. Due to the Au NPs, the carriers are trapped longer than the bare MoS_2_-GaN system, thereby explaining the emission quenching from the GaN bandedge.

**Figure 5:**
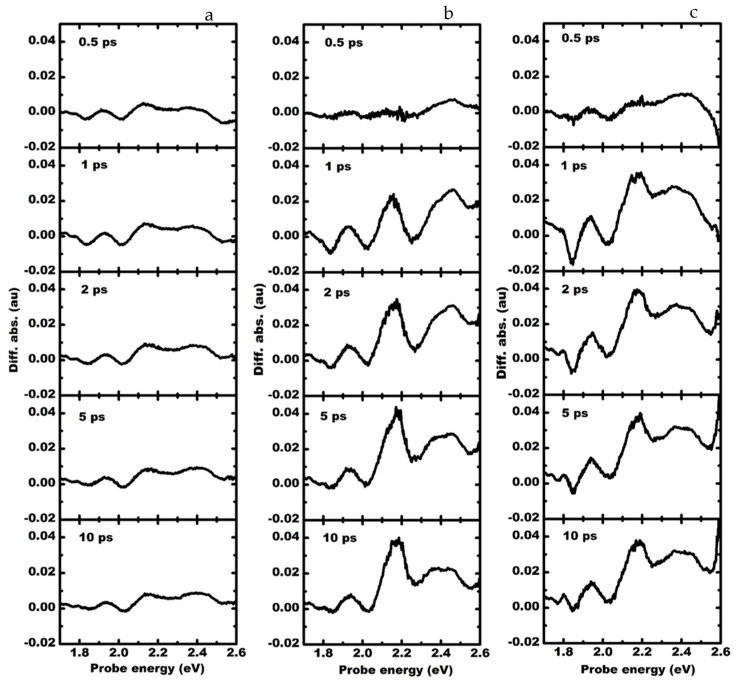
The transient absorption spectrum of (**a**) MoS_2_-GaN (left column), (**b**) MoS_2_-GaN with Au plasmons (middle column), and (**c**) MoS_2_-GaN with Pt plasmons (right column) with 3.54 eV pump at different delay times showing the dynamics of A and B excitonic absorption state in MoS_2_, photoinduced absorption band and the defect band of GaN.

The spectral evolution of the transient differential absorption due to an optical pump at 3.54 eV is shown in Figure 5. In the transient absorption spectrum, the filling of excitonic states gives rise to a decrease in absorption of the probe, followed by the formation of negative amplitude bands, called photobleaching bands. The LSP band due to Pt NPs is broad. Therefore, LSP due to Pt NPs is resonantly coupled to the GaN band-edge and the C excitonic band of MoS_2_. The C band in MoS_2_ is significantly red-shifted in the presence of GaN due to interfacial coupling [6]. In the presence of Pt NPs, the formation of the C band is substantially changed due to the resonant coupling of Pt NPs with GaN and MoS_2_. The C excitonic band gradually redshifts. The amplitude of A and B excitonic bands are higher, and the relaxation of carriers from A and B excitonic bands is faster due to the resonance, as shown by the enhanced recovery of the probe absorption (Supporting Figure S2) compared to MoS_2_ on quartz or in the presence of Au plasmons on MoS_2_ on GaN.

The amplitude of the photobleaching band at 2.26 eV is larger in the presence of Pt nanoparticles than in the presence of Au nanoparticles (Figure 5c). Still, the dynamics of this photobleaching band are almost temporally independent. With 3.54 eV optical excitation, the LSP of Pt resonantly couples with the C exciton band but is off-resonant to the defect band of GaN. However, the A and B excitons in MoS_2_ are strongly influenced by the presence of the Pt plasmons due to the enhanced band edge and defect emission from the GaN. Because of the resonant optical excitation near the Г valley of MoS_2_ and the interband transition in GaN, the density of optically excited carriers with 3.54 eV excitation is quite high. As a result, the amplitude of the Photobleaching bands formed at A and B excitonic bands formed is higher, and the recovery of probe absorption is faster at the A and B exciton positions. Strong photobleaching of the exciton absorption is observed, which occurs within one ps as the probe is completely transparent at 2.26 eV (Figure 4b). After one ps, the magnitude of the photobleaching reduces as the excitons and the plasmons are decoupled, as was reported earlier [11]. However, the dynamics of the defect band are not influenced by the Pt NPs.

The Au NPs are resonant to the defect band of GaN and hence show a remarkable difference in the band’s dynamics at 2.26 eV. The photoinduced absorption of the probe pulse first decreases and becomes negative at a delay time of about 0.8 ps and then gradually increases, as shown in Figure 4b. This behavior is completely different from that of MoS_2_ on GaN or in the presence of Pt NPs. The appearance of plasmon-induced transparency dip and the gradual recovery of the photoinduced absorption is attributed to LSP resonance due to Au NPs. The density of the carriers trapped in the defect band of GaN is higher. Since the recombination lifetime of the carriers at the defect band is much higher, the absorption of the probe initially decreases. The localized plasmon modes due to Au NPs are coupled resonantly with the yellow luminescence band in GaN. The transfer of hot carriers from Au plasmons to the conduction band of MoS_2_ and the trapping of excited carriers in MoS_2_ within the GaN defects results in a further decrease in the absorption of the probe signal. This results in the formation of the transparency dip observed at a delay time of about 1.28 ps after the pump pulse. Once the carriers trapped in the GaN defects relax, the absorption of the probe gradually increases. Similar Au plasmon-induced hot carrier-mediated modulation of exciton absorption has been observed in the MoS_2_ system [25].

**Figure 6:**
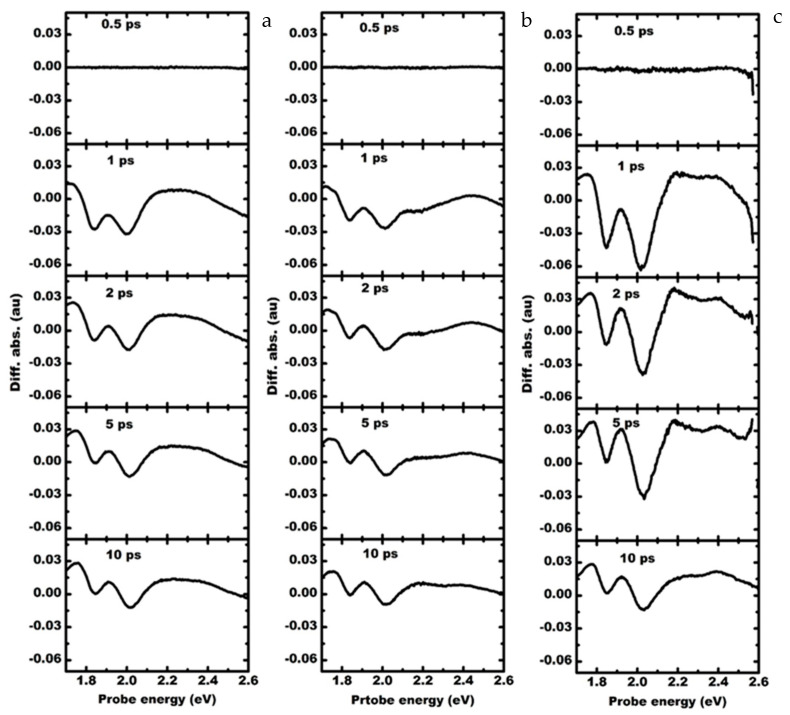
Transient absorption spectrum of (**a**) MoS_2_-GaN (left column), (**b**) MoS_2_-GaN with Au plasmons (middle column) and (**c**) MoS_2_-GaN with Pt plasmons (right column) 3.06 eV pump excitation.

Figure 6 shows the spectral evolution of the transient absorption spectrum with a 3.06 eV excitation. There is a relatively low density of carriers in the defect states, and there is no emission to act as a secondary pump for the excitons. In the case of excitation resonant to the Г bandgap of MoS_2_, the magnitude of the differential transient exciton absorption A and B is significantly enhanced under the influence of localized plasmons due to Pt nanoparticles. The increase in the carrier density in the continuum states resonant to the localized plasmonic field is relaxed rapidly to the A and B states and modifies the exciton absorption. This change is not observed in the bare or Au-plasmon-based MoS_2_-GaN system. It can be observed from Figure 6c that the enhancement in the exciton absorption modulation is observed within 5 ps.

The hybrid integration of GaN to a non-conventional substrate can provide a novel material system. Gallium nitride systems, such as quantum dots or thin films, have been conjugated into oligonucleotides for biophotonic or optoelectronic applications [26,27]. The charge transfer from the organic semiconductors or the self-assembled G-quartets can result in efficient UV detectors or fluorescent markers. Metal nanoparticles attached to graphene-based 2D material, such as graphene oxide, have been used as tunable light emitters [28,29]. The ultrafast modulation of light can lead to Tb/s modulators that are primarily possible through more complex phonon-mediated intersubband transitions in a quantum well [30,31,32] or more recently reported the possibility of using organic-inorganic perovskite materials [33]. The absorption spectrum can be broadened using the plasmonic material in the hybrid MoS_2_-GaN semiconductor system. Using the Pt nanoparticle system combined with the MoS_2_-GaN, the entire UV-Visible range of the electromagnetic spectrum can be efficiently covered for wideband photoabsorption. It can be effectively used for photovoltaic applications. The Au-nanoparticles can modulate the defect level emission. This photoinduced modulation of the defect states can be used for the white light generation that involves the blue light from GaN bandedge states, the green emission from the defect states, and the red emission from the MoS_2_ excitonic states.

In conclusion, the effect of localized plasmon modes resonant to the bandedge, defect states, and the excitonic energy levels of the hybrid MoS_2_/GaN has been studied. The modification of the defect states due to the localized plasmon modes modifies the effective carrier density available for the optical excitation process. It contributes to the modification of the bandedge emission of GaN. Platinum nanoparticles with a broad plasmon resonance in the ultraviolet wavelength range effectively enhance ultraviolet emission at 3.4 eV. For the above GaN bandgap optical excitation, the gold nanoparticles can modify the transient absorption of the defect states in GaN due to their extended lifetime but do not contribute to the light enhancement due to the localized plasmon interaction. The Au localized plasmons’ resonant excitation of the defect states can result in a negative transient decay. Our study is crucial to realize the broadband devices by selectively modulating the electronic density of states of the semiconductor devices.

## Data Availability

Data will be provided on request to the authors.

## Supplementary Materials

The following supporting information can be downloaded at: https://www.mdpi.com/article/10.3390/ma15217422/s1, Figure S1: The effective Absorption spectrum of MoS_2_ on GaN. Figure S2: Decay kinetics probed at (a) A excitonic band and (b) B excitonic band of MoS_2_ for MoS_2_ on quartz (black), MoS_2_-GaN (red), Au-MoS_2_-GaN (blue) and Pt-MoS_2_-GaN (pink) measured with 3.54 eV pump excitation. Figure S3: Decay kinetics probed at (a) A excitonic band and (b) B excitonic band of MoS_2_ for MoS_2_ on quartz (black), MoS_2_-GaN (red), Au-MoS_2_-GaN (blue) and Pt-MoS_2_-GaN (pink) measured with 3.06 eV pump excitation.

## References

[1] Gupta P., Rahman A.A., Subramanian S., Gupta S., Thamizhavel A., Orlova T., Rouvimov S., Vishwanath S., Protasenko V., Laskar M.R. (2016). Layered transition metal dichalcogenides: Promising near-lattice-matched substrates for GaN growth. Sci. Rep..

[2] Morkoc H. (2008). Handbook of Nitride Semiconductors and Devices. Mater. Prop. Phys. Growth.

[3] Neogi A., Morkoç H., Kuroda T., Tackeuchi A. (2005). Coupling of spontaneous emission from GaN–AlN quantum dots into silver surface plasmons. Opt. Lett..

[4] Bilgin I., Liu F., Vargas A., Winchester A., Man M.K.L., Upmanyu M., Dani K.M., Gupta G., Talapatra S., Mohite A.D. (2015). Chemical Vapor Deposition Synthesized Atomically Thin Molybdenum Disulfide with Optoelectronic-Grade Crystalline Quality. ACS Nano.

[5] Poudel Y., Sławińska J., Gopal P., Seetharaman S., Hennighausen Z., Kar S., D’souza F., Nardelli M.B., Neogi A. (2019). Absorption and emission modulation in a MoS2-GaN (0001) heterostructure by interface phonon exciton coupling. Photon. Res. PRJ.

[6] Carvalho A., Ribeiro R.M., Neto A.H.C. (2013). Band nesting and the optical response of two-dimensional semiconducting transition metal dichalcogenides. Phys. Rev. B.

[7] Ghods S., Esfandiar A. (2021). Plasmonic enhancement of photocurrent generation in two-dimensional heterostructure of WSe2/MoS2. Nanotechnology.

[8] Luo J.J., Qin L.Y., Du X.J., Luo H.Q., Li N.B., Li B.L. (2021). Mercury ion-engineering Au plasmonics on MoS_2_ layers for absorption-shifted optical sensors. Anal. Methods.

[9] Li J., Ji Q., Chu S., Zhang Y., Li Y., Gong Q., Liu K., Shi K. (2016). Tuning the photo-response in monolayer MoS2 by plasmonic nano-antenna. Sci. Rep..

[10] Karna S., Mahat M., Choi T.-Y., Shimada R., Wang Z., Neogi A. (2016). Competition Between Resonant Plasmonic Coupling and Electrostatic Interaction in Reduced Graphene Oxide Quantum Dots. Sci. Rep..

[11] Poudel Y., Lim G.N., Moazzezi M., Hennighausen Z., Rostovtsev Y., D’Souza F., Kar S., Neogi A. (2019). Active Control of Coherent Dynamics in Hybrid Plasmonic MoS2 Monolayers with Dressed Phonons. ACS Photonics.

[12] Mahat M. (2018). Phonon-assisted plasmon-induced transparency in reduced graphene oxide. ACS Photonics.

[13] Koya A.N., Zhu X., Ohannesian N., Yanik A.A., Alabastri A., Zaccaria R.P., Krahne R., Shih W., Garoli D. (2021). Nanoporous Metals: From Plasmonic Properties to Applications in Enhanced Spectroscopy and Photocatalysis. ACS Nano.

[14] Ozgur U., Lee C.W., Everitt H. (2000). Control of Coherent Acoustic Phonons in Semiconductor Quantum Wells. Phys. Rev. Lett..

[15] Koya A.N., Cunha J., Guerrero-Becerra K.A., Garoli D., Wang T., Juodkazis S., Zaccaria R.P. (2021). Plasmomechanical Systems: Principles and Applications. Adv. Funct. Mater..

[16] Lee I.-H., Polyakov A.Y., Yakimov E.B., Smirnov N.B., Shchemerov I.V., Tarelkin S.A., Didenko S.I., Tapero K.I., Zinovyev R.A., Pearton S.J. (2017). Defects responsible for lifetime degradation in electron irradiated n-GaN grown by hydride vapor phase epitaxy. Appl. Phys. Lett..

[17] Li L., Yu J., Hao Z., Wang L., Wang J., Han Y., Li H., Xiong B., Sun C., Luo Y. (2017). Influence of point defects on optical properties of GaN-based materials by first principle study. Comput. Mater. Sci..

[18] Lu S.-C., Leburton J.-P. (2014). Electronic structures of defects and magnetic impurities in MoS2 monolayers. Nanoscale Res. Lett..

[19] Lee E.W., Lee C.H., Paul P.K., Ma L., McCulloch W.D., Krishnamoorthy S., Wu Y., Arehart A.R., Rajan S. (2015). Layer-transferred MoS_2_/GaN PN diodes. Appl. Phys. Lett..

[20] Liao J., Ji L., Zhang J., Na Gao N., Li P., Huang K., Yu E.T., Kang J. (2018). Influence of the Substrate to the LSP Coupling Wavelength and Strength. Nanoscale Res. Lett..

[21] Liang L., Meunier V. (2014). First-principles Raman spectra of MoS2, WS2 and their heterostructures. Nanoscale.

[22] Davydov V.Y., Kitaev Y.E., Goncharuk I.N., Smirnov A.N., Graul J., Semchinova O., Uffmann D., Smirnov M.B., Mirgorodsky A.P., Evarestov R.A. (1998). Phonon dispersion and Raman scattering in hexagonal GaN and AlN. Phys. Rev. B.

[23] Neogi A., Gryczynski K., Llopis A., Lin J., Main K., Shimada R., Wang Z., Lee J., Salamo G., Krokhin A. (2016). Metallic Nanodroplet Induced Coulomb Catalysis for Off-Resonant Plasmonic Enhancement of Photoemission in Semiconductors. ACS Omega.

[24] Shi H., Yan R., Bertolazzi S., Brivio J., Gao B., Kis A., Jena D., Xing H.G., Huang L. (2013). Exciton Dynamics in Suspended Monolayer and Few-Layer MoS_2_ 2D Crystals. ACS Nano.

[25] Li Z., Xiao Y., Gong Y., Wang Z., Kang Y., Zu S., Ajayan P.M., Nordlander P., Fang Z. (2015). Active Light Control of the MoS2 Monolayer Exciton Binding Energy. ACS Nano.

[26] Neogi A., Li J., Neogi P., Sarkar A., Morkoc H. (2004). Self-assembled modified deoxyguanosines conjugated to GaN quantum dots for biophotonic applications. Electron. Lett..

[27] Liddar H., Li J., Neogi A., Neogi P.B., Sarkar A., Cho S., Morkoç H. (2008). Self-assembled deoxyguanosine based molecular electronic device on GaN substrates. Appl. Phys. Lett..

[28] Neogi A., Karna S., Shah R., Philipose U., Perez J., Shimada R., Wang Z.M. (2014). Surface plasmon enhancement of broadband photo-luminescence emission from graphene oxide. Nanoscale.

[29] Walker E., Reyes D., Rojas M.M., Krokhin A., Wang Z., Neogi A. (2014). Tunable ultrasonic phononic crystal controlled by infrared radiation. Appl. Phys. Lett..

[30] Neogi A., Yoshida H., Mozume T., Wada O. (1999). Enhancement of interband optical nonlinearity by manipulation of intersubband transitions in an undoped semiconductor quantum well. Opt. Commun..

[31] Neogi A., Yoshida H., Mozume T., Georgiev N., Wada O. (2001). Intersubband transitions and ultrafast all-optical modulation using multiple InGaAs-AlAsSb-InP coupled double-quantum-well structures. IEEE J. Sel. Top. Quantum Electron..

[32] Neogi A., Takahashi Y., Kawaguchi H. (1996). Interband nonlinear optical generation in the presence of intersubband light in asym-metric quantum wells. IEEE J. Quantum Electron..

[33] Grinblat G., Abdelwahab I., Nielsen M.P., Dichtl P., Leng K., Oulton R.F., Loh K.P., Maier S.A. (2019). Ultrafast All-Optical Modulation in 2D Hybrid Perovskites. ACS Nano.

