# Supplementary material for: Plasmon-Induced Enhanced Light Emission and Ultrafast Carrier Dynamics in a Tunable Molybdenum Disulfide-Gallium Nitride Heterostructure
Source: Materials (Basel). 2022 Oct 22;15(21):7422. doi: 10.3390/ma15217422 (PMC9657517; doi:10.3390/ma15217422)
Supplement: Supplementary file 1 [file materials-15-07422-s001.zip › materials-1932231-supplementary.pdf]

materials

Supplementary Document

S1: Steady-State or Equilibium Absorption Characteristics:

The multilayer MoS_2 on the GaN template was characterized before the nucleation of the metal nanoparticles. This first figure of the supplementary document shows the steady-state absorption characteristics measured using UV-enhanced white light. The room temperature excitonic transitions are clearly observed at 1.9 eV and 2.1 eV. One can observe a rise in the absorption edge above 3.3 eV at the higher energy side.

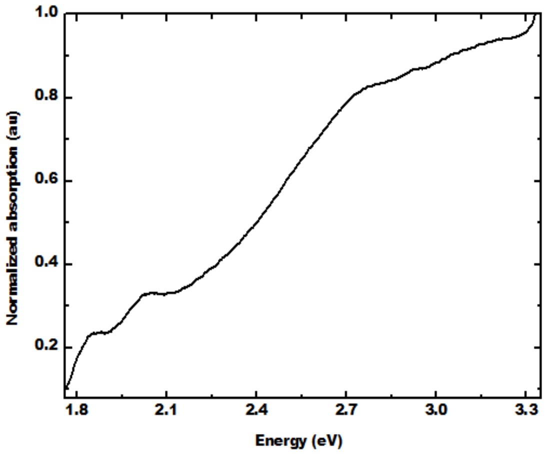

Figure S1. The effective Absorption spectrum of MoS_2 on GaN.

S2. Decay Kinetics of A and B excitons

(a) with optical excitation above GaN bandgap and resonant to Pt nanoparticles:

The time-dependent change in the differential absorption due to optical pumping at 3.54 eV is plotted at the A and B exciton states of MoS_2. Although the Au nanoparticles with plasmon energy at 2.26 eV are not directly excited by the optical pumping, their resonance with the defect states of GaN centered at 2.25 induces the most significant change in the A exciton's transmission. The rise and decay times for the A excitons are not significantly different for either Au or Pt plasmons system. However, the magnitude of the change is significantly higher for the plasmons due to Au nanoparticles than the reference samples or the Pt-induced plasmons. In the case of the B excitons, the Au plasmons induce a significantly faster absorption recovery rate.

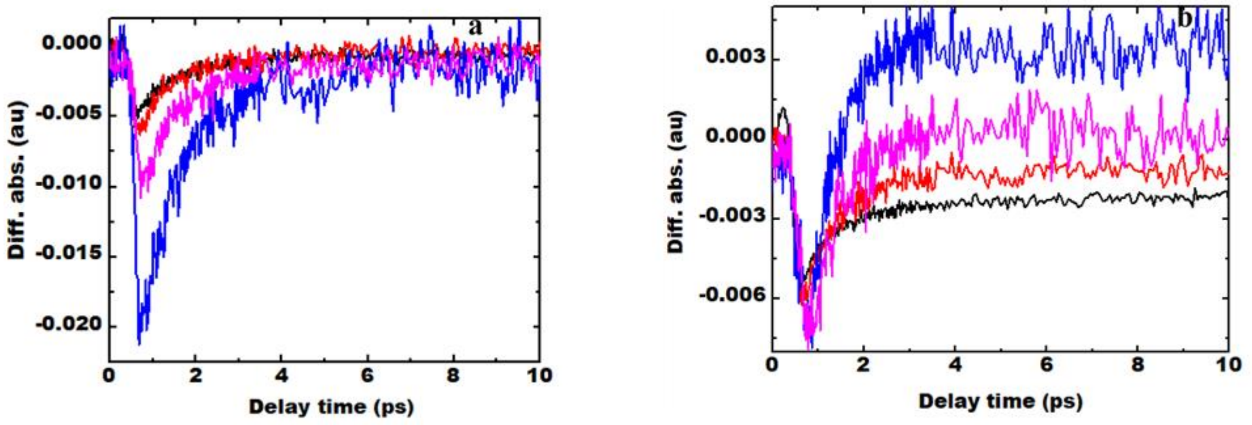

Supporting Figure S2. Decay kinetics probed at (a) A excitonic band and (b) B excitonic band of MoS_2 for MoS_2 on quartz (black), MoS_2-GaN (red), Au-MoS_2-GaN (blue) and Pt-MoS_2-GaN (pink) measured with 3.54 eV pump excitation.

(b) with optical excitation below GaN bandgap and resonant to C-exciton states

It has been observed in previous work [18] that carriers excited to the continuum states due to an optical excitation of the C-exciton states can relax to A and B exciton states within a picosecond. The presence of the LSP modes Au increases the stronger absorption change and for the A exciton, this absorption recovery is faster compared to Pt nanoparticles. However, in the case of the B exciton, the carrier relaxation does not completely recover and has a relatively long-lived lifetime.

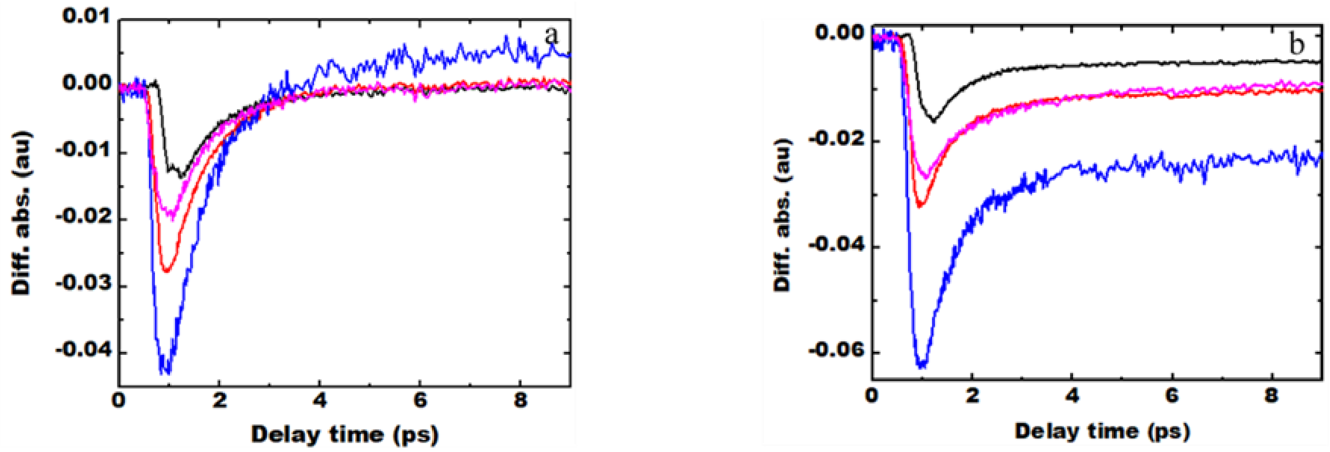

Supporting Figure S3. Decay kinetics probed at (a) A excitonic band and (b) B excitonic band of MoS_2 for MoS_2 on quartz (black), MoS_2-GaN (red), Au-MoS_2-GaN (blue) and Pt-MoS_2-GaN (pink) measured with 3.06 eV pump excitation.
